# Targeted next-generation-sequencing for reliable detection of targetable rearrangements in lung adenocarcinoma—a single center retrospective study

**DOI:** 10.1016/j.prp.2018.02.001

**Published:** 2018-04

**Authors:** Nadezda P. Velizheva, Markus P. Rechsteiner, Nadejda Valtcheva, Sandra N. Freiberger, Christine E. Wong, Bart Vrugt, Qing Zhong, Ulrich Wagner, Holger Moch, Sven Hillinger, Isabelle Schmitt-Opitz, Alex Soltermann, Peter J. Wild, Verena Tischler

**Affiliations:** a Institute of Pathology and Molecular Pathology, University Hospital Zurich, Zurich, Switzerland; b Department of Thoracic Surgery, University Hospital Zurich, Zurich, Switzerland; c Dr. Senckenberg Institute of Pathology, University Hospital Frankfurt, Frankfurt am Main, Germany

**Keywords:** Next generation sequencing, Lung adenocarcinoma, Gene fusion, *ALK* gene

## Abstract

Oncogenic rearrangements leading to targetable gene fusions are well-established cancer driver events in lung adenocarcinoma. Accurate and reliable detection of these gene fusions is crucial to select the appropriate targeted therapy for each patient. We compared the targeted next-generation-sequencing Oncomine Focus Assay (OFA; Thermo Fisher Scientific) with conventional *ALK* FISH and anti-Alk immunohistochemistry in a cohort of 52 lung adenocarcinomas (10 *ALK* rearranged, 18 non-*ALK* rearranged, and 24 untested cases). We found a sensitivity and specificity of 100% for detection of *ALK* rearrangements using the OFA panel. In addition, targeted next generation sequencing allowed us to analyze a set of 23 driver genes in a single assay. Besides *EML4-ALK* (11/52 cases), we detected *EZR-ROS1* (1/52 cases), *KIF5B-RET* (1/52 cases) and *MET-MET* (4/52 cases) fusions. All *EML4-ALK*, *EZR-ROS1* and *KIF5B-RET* fusions were confirmed by multiplexed targeted next generation sequencing assay (Oncomine Solid Tumor Fusion Transcript Kit, Thermo Fisher Scientific). All cases with *EML4-ALK* rearrangement were confirmed by Alk immunohistochemistry and all but one by *ALK* FISH. In our experience, targeted next-generation sequencing is a reliable and timesaving tool for multiplexed detection of targetable rearrangements. Therefore, targeted next-generation sequencing represents an efficient alternative to time-consuming single target assays currently used in molecular pathology.

## 1 Introduction

Since its discovery in 2007, oncogenic *EML4-ALK* rearrangements have been intensively studied in lung cancer biology and therapy [[1], [2], [3], [4]]. Meanwhile, first line Alk kinase inhibitor therapy with crizotinib is the current standard of care in *ALK* rearranged lung adenocarcinoma (LUAD) with increased progression-free survival compared with conventional chemotherapy [5]. Therefore, a reliable and accurate detection of such *ALK* rearrangements is essential for the molecular pathology workflow. Approximately 3–7% of LUADs harbor *ALK* rearrangements in Caucasian populations. Histological morphology of *ALK* rearranged LUAD is typically solid with few foci of signet ring cells [3]. Other cancer driver fusion genes in LUAD are *ROS1* and *RET* [[6], [7], [8]]. The resulting chimeric proteins also are therapeutic targets [[9], [10], [11]]. *ROS1* rearrangements are found in approximately 2% of LUADs and *RET* rearrangements in 1%, respectively. *MET* splice site mutations resulting in exon 14 skipping and activation of the c-Met pathway occur in approximately 4% of LUADs [12,13]. Patients with these mutations were shown to respond to MET inhibition [14]. Multiplexed assays like targeted next-generation-sequencing (NGS) approaches allow the analysis of large set of genomic alterations compared with single target assays like fluorescence *in-situ* hybridization (FISH) and immunohistochemistry (IHC). We have previously demonstrated the feasibility and reliable application of DNA- and RNA- based targeted sequencing in a cohort of small tissue samples and cytological specimens [15]. The aim of the present study was to investigate the performance of a RNA-based targeted NGS assay for detection of targetable fusion genes and compare the results with corresponding FISH and IHC assays.

## 2 Materials and methods

### 2.1 Patient samples and cell lines

We tested a cohort of advanced lung adenocarcinomas (LUADs) (n = 52) in this retrospective validation study (Table 1). Formalin-fixed paraffin-embedded (FFPE) LUAD tissue blocks were collected from our archives between 2003 and 2008. All samples were processed according to National Comprehensive Cancer Network (NCCN) and Swiss Society of Pathology (SSPath) guidelines. Tumor cell content was assessed by board-certified pathologists on a multi-headed microscope (VT, AS, BV). Only unambiguous LUAD samples with tumor cell content ≥ 60% were included in the study cohort. Among included LUAD cases, 10 samples were *ALK* rearranged, 18 cases were non-*ALK* rearranged as detected by fluorescence *in-situ* hybridization (FISH). The remaining 24 cases had not been tested before (Table 1). The study was approved by the Cantonal Ethics Committee of Zurich (StV-No. 2009-0029 and KEK-ZH-No. 2014-0604).

**Table 1:** LUAD samples included in the study cohort (n = 52).

*ALK* status	Sample No.
Positive, n = 10	1–10
Negative, n = 18	11–20, 32, 36, 42, 43, 45, 46, 51, 52
Unknown, n = 24	21–31, 33–35, 37–41, 44, 47–50

*ALK*, anaplastic lymphoma kinase.

H3122 (*EML4-ALK* rearranged) and HCC-44 (no *EML4-ALK* rearrangement) were grown in RPMI 1640 medium (Thermo Fisher Scientific, Carlsbad, CA) with 10% FBS at 37 °C in humidified atmosphere with 5% CO_2_ to 70% confluency. The cells were harvested after rinsing with phosphate buffered saline using 0.25% trypsine (Thermo Fisher Scientific, Carlsbad, CA). Cells were washed in RPMI medium, pelleted and formalin fixated. For cell blocks, protein glycerol (Morphisto GmbH, Frankfurt) clotting followed by routine histological processing was performed.

### 2.2 RNA extraction

We extracted RNA from three tissue cores (diameter 0.6 mm) punched from the formalin-fixed paraffin-embedded (FFPE) tissue blocks or from FFPE sections of the cell blocks from cell lines. Normal tissue was not analyzed. Tumor tissue cylinders were deparaffinized with 1000 μl xylene and washed twice in 800 μl ethanol. After drying at 37 °C the samples were digested with proteinase K at 56 °C overnight. To avoid genomic DNA contamination, samples were treated with DNase1 for 15 min at room temperature (RT). RNA extraction was performed applying the Maxwell 16 LEV RNA FFPE Purification Kit (Promega). RNA was quantified with Qubit 2.0 using the RNA HS Assay Kit (Thermo Fisher Scientific). We assessed RNA quality with the Agilent 2100 Bioanalyzer (Agilent Technologies, Basel, Switzerland).

### 2.3 Targeted NGS

Targeted RNA-based NGS of LUADs has already been performed on a cohort of small biopsies and cytology smears at the Institute of Pathology and Molecular Pathology [15]. Targeted RNA-based NGS was conducted with the Oncomine Focus Assay (OFA) panel (Thermo Fisher Scientific, Carlsbad, CA) which is designed to detect gene fusions involving 23 fusion drivers (*ABL1, ALK, AKT3, AXL, BRAF, EGFR, ERBB2, ERG, ETV1, ETV4, ETV5, FGFR1, FGFR2, FGFR3, MET, NTRK1, NTRK2, NTRK3, PDGFRA, PPARG, RAF1, RET, ROS1*).

We validated the OFA results with the Oncomine Solid Tumor Fusion Transcript Kit (Thermo Fisher Scientific), another targeted NGS assay to detect relevant gene fusions (*ALK, ROS1, RET, NTRK*). All libraries were prepared using 10 ng of starting RNA. RNA was first reverse transcribed with the Invitrogen SuperScript VILO cDNA Synthesis Kit (Thermo Fisher Scientific). The resulting cDNA was used as input for the targeted amplification. After targeted amplification with the corresponding panel, all libraries were labeled with Ion Xpress™ Barcode Adapters (Thermo Fisher Scientific). Libraries were quantified and mixed according to manufacturer’s recommendations. The Ion Hi-Q Chef Kit and the Ion Chef System were used for template preparation and enrichment. Enriched libraries were then loaded on the Ion 318 Select Chip and sequenced on the Ion PGM System (Thermo Fisher Scientific) using the Ion PGM Hi-Q Sequencing Kit (Thermo Fisher Scientific). The detection of a rearrangement in a sample was judged as true positive following the manufacturer’s recommendations (Thermo Fisher Scientific). Statistics and sequencing data analysis were performed as described [15]. Visualization of detected fusion events was made using Integrative Genomics Viewer (IGV; Broad Institute, Cambridge, MA) demonstrating the alignment of sequenced reads to the reads of known fusion breakpoints and the reference human genome hg19.

### 2.4 Fluorescence *in-situ* hybridization (FISH)

For testing of *ALK, ROS1,* and *RET* rearrangements, the Abbott Molecular/Vysis LSI ALK Break Apart Rearrangement Probe (Abbott Molecular, Baar, Switzerland), Zyto*Light* SPEC ROS1 Dual Color Break Apart Probe (Zytovision GmbH, Bremerhaven, Germany), and Zyto*Light* SPEC RET Dual Break Apart Probe (Zytovision GmbH, Bremerhaven, Germany) were applied. FISH testing was performed on whole sections of LUAD specimens. For each particular case, a board certified pathologist analyzed 100 tumor nuclei. A sample was called true positive if  ≥15% of tumor nuclei showed split signals according to the manufacturer’s evaluation guidelines (Abbott Molecular, Des Plaines, IL). A second pathologist independently counted borderline cases.

### 2.5 Immunohistochemistry (IHC)

Alk and Ros1 IHC was performed on 0.6 mm tissue cylinders as previously described [16,17]. For Alk IHC, the mouse anti-human ALK monoclonal antibody was applied (clone 5A4, Leica Biosystems). Ros1 IHC was conducted using a rabbit anti-human ROS1 monoclonal antibody (clone D4D6, Cell Signaling Technology). For c-Met IHC a monoclonal rabbit anti-human Met antibody was used (clone SP44, Spring Biosciences). All buffers, including pretreatment CC1 standard incubation buffer, secondary antibody (UltraMap anti-Rabbit HRP) and detection system Discovery ChromoMap DAB, were purchased from Roche Ventana (Tucson, AZ). Immunostainings were performed on the automated immunostainer DiscoveryUltra (Roche Ventana).

## 3 Results

### 3.1 RNA metrics

We examined 52 LUAD cases, of which 10 had *ALK* rearrangement confirmed by FISH and immunohistochemistry (Table 1). Eighteen cases were confirmed non-*ALK* rearranged samples by FISH and immunohistochemistry (Table 1). The remaining 24 cases had an unknown *ALK* status (Table 1). The extracted RNA of the three FFPE tissue cores per case showed an average RNA concentration of 48 ng/μl (median 42.2 ng/μl, standard deviation ± 29.5 ng/μl, range 11.2–152 ng/μl) and a low RNA integrity number (RIN) of 1.1–2.6 (13 cases RIN not available, Table 2). The fragment length >150 bp ranged from 22 to 90% (13 cases not available, Table 2). All RNA samples were processed using two assays, the Oncomine Focus Assay (OFA) and the Oncomine Solid Tumor Fusion Transcript Kit, respectively. For the OFA panel, the total mapped fusion panel reads ranged from 25,705–269,517 (Table 2). Amplification of the 5 control genes was successful in all cases. The number of total mapped fusion panel reads (TMFPR) was not correlated with the RIN (correlation coefficient 0.108, p-value 0.5139) or with the percentage of fragments >150 bp (correlation coefficient 0.268, p-value 0.09).

**Table 2:** Metrics of extracted RNA and NGS parameters of the Oncomine Focus Assay panel.

Sample No.	Quantification	Quality		NGS metrics		
	Qubit (ng/μl)	RIN	RNA fragments >150 base pairs (%)	TMFPR	Gene (Exons)	Read Counts
1	27	2.2	67	194460	*EML4(6) – ALK(20)*	527
2	29.8	2.5	46	106905	*EML4(6) – ALK(20)*	518
3	152	NA	NA	38816	*EML4(6) – ALK(20)*	632
4	11.2	2.5	51	199487	*EML4(6) – ALK(20)*	2277
5	11.3	2.4	59	194261	*EML4(6) – ALK(20)*	370
6	12.4	2.5	38	137627	*EML4(13) – ALK(20)*	3108
7	40	NA	NA	49725	*EML4(6) – ALK(20)*	574
8	18.3	NA	NA	68458	*EML4(13) – ALK(20)*	3294
9	26	NA	NA	42774	*EML4(6) – ALK(20)*	1790
10	20.6	NA	NA	27414	*EML4(6) – ALK(20)*	533
11	14.7	2.3	67	179102	*EZR(10) – ROS1(34)*	8981
12	44.2	2.4	24	148025	no fusion	–
13	42.6	1.1	22	151081	no fusion	–
14	49.8	NA	NA	41152	no fusion	–
15	24.4	NA	NA	63798	no fusion	–
16	52.2	NA	NA	196200	no fusion	–
17	35.4	NA	NA	230033	no fusion	–
18	47	NA	NA	131974	no fusion	–
19	35.4	2.3	60	269517	no fusion	–
20	34.8	2.5	47	99363	no fusion	–
21	64.4	2.4	68	114466	no fusion	–
22	76.6	2.3	73	186135	no fusion	–
23	66.4	2.4	49	84206	no fusion	–
24	30.6	2.5	46	135882	no fusion	–
25	23.2	2.5	42	38390	no fusion	–
26	37.2	2.4	52	150894	no fusion	–
27	22.2	2.5	46	172565	no fusion	–
28	21	2.5	43	192538	no fusion	–
29	40.6	2.5	40	114472	no fusion	–
30	63	2.3	63	253671	*EML4(13) – ALK(20)*	2626
31	87.2	2.3	64	183584	no fusion	–
32	50.8	2.4	51	234307	no fusion	–
33	19.6	1.6	76	35200	no fusion	–
34	55.8	2.4	57	215167	no fusion	–
35	83.6	2.3	58	196873	no fusion	–
36	74.8	2.4	57	237336	*KIF5B(15) – RET(12)*	14257
37	50.8	2.4	65	169419	no fusion	–
38	73	2.4	54	131217	no fusion	–
39	61.6	2.3	65	183111	no fusion	–
40	130	2	74	114982	no fusion	–
41	26.6	2.2	38	25705	no fusion	–
42	26.4	2.4	52	188899	no fusion	–
43	50.4	2.4	62	206721	no fusion	–
44	50.8	2.5	43	169713	no fusion	–
45	25.6	2.6	37	102602	no fusion	–
46	80	2.4	50	119869	no fusion	–
47	56.4	2.2	58	206807	no fusion	–
48	100	2.1	69	175943	no fusion	–
49	30.8	2.3	90	181785	*EZR(10) – ROS1(34)*	76
50	41.8	NA	NA	95416	no fusion	–
51	100	NA	NA	143052	no fusion	–
52	43.6	NA	NA	59579	no fusion	–

Legend: RIN, RNA integrity number; NGS, next-generation sequencing; TMFPR, total mapped fusion panel reads; NA, not assessed; EML4-ALK, echinoderm microtubule-associate protein-like 4-anaplastic lymphoma kinase; EZR-ROS1, ezrin gene-proto-oncogene tyrosine-protein kinase 1; KIF5B-RET, the kinesin family 5 B gene-ret proto-oncogene.

### 3.2 Detected gene fusions and performance of NGS based assays

**Fig. 1:**
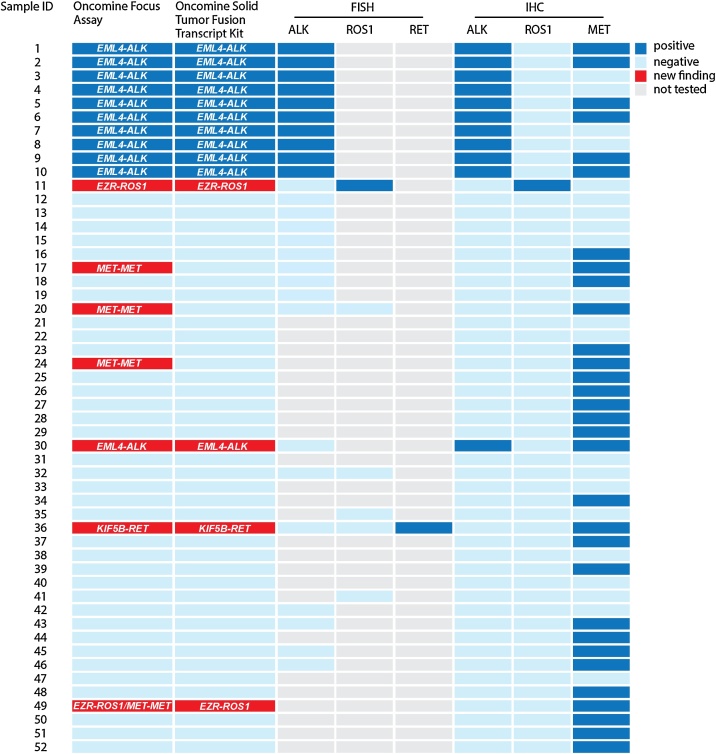
Genetic rearrangements in study cohort of LUADs. Overview of NGS results compared to FISH and IHC. LUAD lung adenocarcinoma; NGS, next-generation-sequencing; FISH, fluorescence *in-situ* hybridization; IHC, immunohistochemistry; *EML4-ALK,* echinoderm microtubule-associate protein-like 4 − anaplastic lymphoma kinase; *EZR-ROS1,* ezrin gene − proto-oncogene tyrosine-protein kinase 1; *KIF5B-RET,* the kinesin family 5 B gene-ret proto-oncogene.

**Fig. 2:**
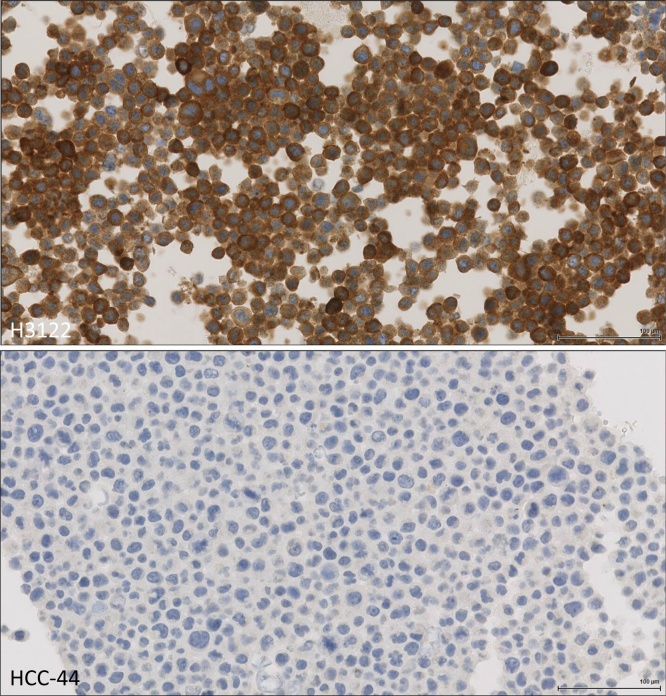
Immunohistochemical staining for Alk, upper tile H3122, lower tile HCC-44.

*EML4-ALK* fusion gene variant 1 with *EML4* exon 13 being fused to *ALK* exon 20 was found in 3/11 (27%) cases and variants 3a/b with *EML4* exon 6 being fused to *ALK* exon 20 were detected in 8/11 (72%) cases (Table 2). Variant 2 with *EML4* exon 20 being fused to *ALK* exon 20 was not detected in our cohort. Other fusion genes than *EML4-ALK*, *EZR-ROS1*, *KIF5B-RET* and *MET-MET* were not detected in our LUAD cohort. We detected one additional *EML4-ALK fusion*, one *EZR-ROS1* fusion and a single *KIF5B-RET* fusion in our LUAD cohort (Fig. 1). Another *EZR-ROS1* fusion (case 49) was detected at a very low read number. However, Ros1 IHC was negative, so that the genomic event could not be confirmed on expression level. The Oncomine Solid Tumor Fusion Transcript Kit confirmed all *EML4-ALK*, *EZR-ROS1* and *KIF5B-RET* events (Fig. 1). In addition, 4 cases of *MET-MET* fusion, resulting in *MET* exon 14 skipping were detected by the OFA and confirmed by c-Met overexpression (Fig. 1). One of the *MET-MET* fusion genes co-occurred with an *EZR-ROS1* fusion (case 49, Fig. 1). For determination of the sensitivity of RNA-based targeted sequencing we compared different ratios of the *EML4-ALK* rearranged cell line H3122 on a background of the non-*EML4-ALK* rearranged cell line HCC-44 (Fig. 2). The lowest detectable ratio was 0.01 with 159 gene specific reads (Table 4).

**Table 4:** NGS metrics of different ratios of *EML4-ALK* to non-*EML4-ALK* rearrangement containing RNA.

Ratio RNA H3122/HCC-44	NGS metrics			
	TMFPR	Gene (Exons)	Read Counts	Ratio Read Counts/TMFPR
1	236702	*EML4(6) – ALK(20)*	42452	0.179347872
0.1	238830	*EML4(6) – ALK(20)*	3067	0.01284177
0.05	197984	*EML4(6) – ALK(20)*	1999	0.010096775
0.01	155527	*EML4(6) – ALK(20)*	159	0.001022331
0.005	270342	*EML4(6) – ALK(20)*	0	0
0.001	234415	*EML4(6) – ALK(20)*	0	0
0.0005	214911	*EML4(6) – ALK(20)*	0	0
0	167480	*EML4(6) – ALK(20)*	0	0

### 3.3 Comparison of targeted NGS with FISH and IHC

**Fig. 3:**
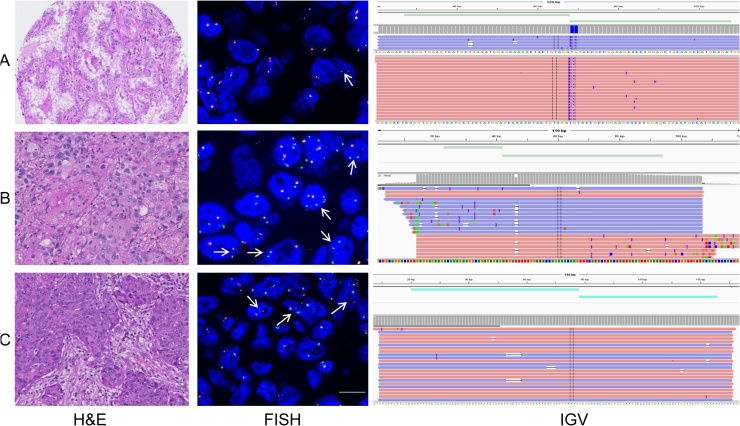
**3A** Shows a newly identified LUAD case with *EML4-ALK* fusion found by OFA and Oncomine Solid Tumor Fusion Transcript Kit assay and confirmed by ALK immunohistochemistry. The FISH assay did not reveal enough break-apart signals required for classification as *ALK* rearrangend. **3B** Newly detected *EZR-ROS1* fusion in a case tested negative for *ALK* FISH and ALK IHC. Both OFA and Oncomine Solid Tumor Fusion Transcript Kit assay as well as retrospective *ROS1* FISH and ROS1 IHC confirmed the alteration. **3C** shows a *RET* fusion confirmed by OFA, Oncomine Solid Tumor Fusion Transcript Kit assay and *RET* FISH.

All 10 known *ALK* fusions and 18 non-rearranged cases were detected by OFA and Oncomine Solid Tumor Fusion Transcript Kit assay (sensitivity 100%, specificity 100%, positive predictive value 100%, negative predictive value 100%, Table 3). In addition, we found one *EML4-ALK* rearranged case (ID 30) within the set of the 24 previously non-tested cases (Fig. 3A). Interestingly, retrospective Alk immunohistochemistry of the same case was positive whereas *ALK* FISH analysis by an investigator blinded for the NGS results revealed 14% of nuclei with break-apart signals, slightly below the threshold of 15% break-apart signals required to confirm *ALK* rearranged LUAD. Reanalysis by an independent second pathologist confirmed the borderline nature of the case.

**Table 3:** Accuracy of NGS-based targeted RNA-sequencing in detection of *ALK* and *ROS1* rearrangements.

*ALK rearrangements*		
	Condition positive	Condition negative
Test positive	11 (TP)	0 (FP)
Test negative	0 (FN)	18 (TN)
	Sensitivity 100%	Specificity 100%
*ROS1* rearrangements		
	Condition positive	Condition negative
Test positive	1 (TP)	1 (FP)
Test negative	0 (FN)	51 (TN)
	Sensitivity 100%	Specificity 98%

*ALK*, anaplastic lymphoma kinase; TP, true positive; FP, false positive; FN, false negative; TN, true negative; ROS1, proto-oncogene tyrosine-protein kinase 1.

To confirm *ROS1* rearrangement detected by OFA and the Oncomine Solid Tumor Fusion Transcript Kit assay all *ROS1* positive cases were further referred to FISH and IHC. Case no. 11 was ROS1 positive when tested by FISH and IHC (Fig. 3B). The second case (ID 49) was negative for ROS1 IHC. Unfortunately, due to lack of tissue resources, *ROS1* FISH analysis could not be performed. We therefore classified this result as false positive (Table 3). One case with *KIF5B-RET* fusion identified by both assays, the OFA and the Oncomine Solid Tumor Fusion Transcript Kit assay (Fig. 3C), showed a break-apart signal in a retrospective *RET* FISH.

All four samples with *MET* exon 14 skipping stained positive with c-Met IHC. In addition, we found c-Met overexpression in 27 of the remaining cases, indicating additional other mechanisms resulting in c-Met overexpression. All *MET(13)-MET(15)* fusion cases had high numbers ( > 200) of specific *MET* reads and sufficient total mapped fusion panel reads.

### 3.4 Variation of fusion read counts

**Fig. 4:**
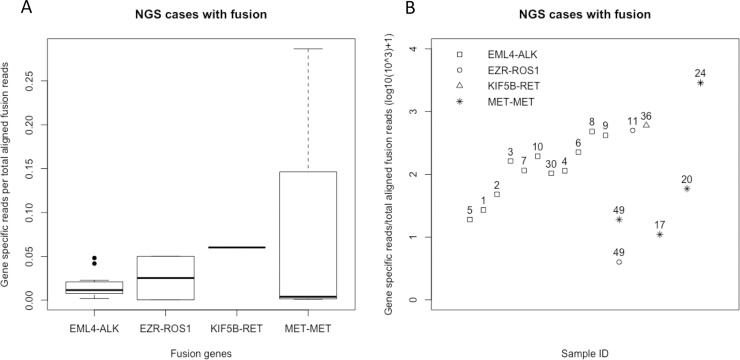
**4A** Boxplots of gene specific reads per total aligned fusion reads for each identified fusion genes (absolute values). **4B**. Plot showing the Sample ID, genomic alteration and gene specific reads per total aligned fusion reads (log10(10^3) + 1).

We observed gene specific read counts ranging from 76 to 38946 for all detected gene fusions. The ratio of gene specific read counts and total mapped fusion panel reads ranged from 0.0004–0.2866. Fig. 4A shows boxplots of read counts for all detected fusion genes. Sample no. 49 had low read counts of 76 for *ROS1*. *EZR-ROS1* rearrangement was confirmed with Oncomine Solid Tumor Fusion Transcript Kit assay. However, Ros1 IHC was repeatedly negative, indicating that the low number of fusion transcripts did not result in a detectable amount of endogenous Ros1 protein with standard Ros1 IHC protocol. Fig. 4 B summarizes the ratios of gene specific reads per total aligned fusion reads.

## 4 Discussion

We studied the performance of a RNA-based targeted sequencing approach versus the gold standard FISH analysis for detection of *ALK* fusions in a retrospective cohort of LUADs. All previously known *ALK* fusions could be detected by targeted NGS. In addition, we found an additional *EZR-ROS1* fusion in one of the *ALK* non-rearranged cases and another potential *EZR-ROS1* fusion in the untested cohort. We also found one additional *EML4-ALK* fusion in the untested cohort.

Our results are concordant with findings by other groups. Pfarr et al. also reported a sensitivity and specificity of 100% of a targeted massively parallel sequencing approach for the detection of known targetable gene fusions [18]. Paasinen-Sohns et al. compared in their study the overall performance of DNA and RNA-based OFA panels and could confirm all detected *EML4-ALK*, *EZR-ROS1* and *MET-MET* fusions with IHC in their cohort [19]. Another study investigating FISH, IHC and NGS regarding *EML4-ALK* rearrangements found 4 tumors positive by FISH and 8 positive by IHC in a cohort of 51 LUAD patients [20]. Of these, only 3 cases were positive by both FISH and IHC [20]. Moreover, 4 of 5 IHC positive and FISH negative patient tumors could be confirmed being *EML4-ALK* rearranged by NGS [20]. Two of these patients with *EML4-ALK* rearranged tumors confirmed by NGS received crizotinib treatment with durable progression-free survival suggesting that especially for borderline cases NGS can tip the scales [20]. A further study was focused at solving discordant ALK testing results determined by IHC and FISH with NGS [21]. NGS testing revealed three *ALK* FISH positive Alk IHC negative cases as *ALK* non-rearranged [21]. Interestingly, Alk immunohistochemistry with ALK1 clone was positive in 12 *ALK* FISH negative cases but turned out to be false positive when Alk IHC was repeated with clone D5F3 which we also used for our study [21].

The recommended cut-offs for FISH analyses remain controversial and might explain the false positive and negative results when comparing FISH with other assays such as NGS. Seventy four per cent of patients with advanced *ALK* rearranged LUAD as determined by FISH responded to first line crizotinib treatment [5]. The median progression free survival was 10.9 months [5]. False negative test results, however, make it impossible to identify patients who might benefit from targeted therapy. False positive results will exclude the patient from further molecular testings in hierarchical single target assays. In addition, false positive results lead to false hope and assignment to wrong therapeutic strategies. The *ALK* reads per total aligned fusion reads of our NGS positive *ALK* FISH negative case no. 30 match the median of all detected *EML4-ALK* cases (0.01) so that a low *ALK* read ratio reflecting a very low transcript number can be ruled out. NGS results for this particular case also fit to the observation that Alk IHC was positive. To further rule out a lack of sensitivity we performed a dilution series of *ALK* rearranged cell line RNA on a non-*ALK* rearranged RNA background. Ratios of 1, 0.1, 0.05, and 0.01 could be detected with RNA-based targeted sequencing. This means that even 1% of *EML4-ALK* rearranged RNA or 1% of *EML4-ALK* rearranged cells can be detected on a background without *EML4-ALK* rearrangement. This also explains the discrepancy of the case being negative according to *EML4-ALK* FISH evaluation criteria but positive by Alk IHC and RNA-based targeted sequencing. Case 30 could harbor a heterogeneous *EML4-ALK* rearrangement so that the rearrangement detection can be difficult by FISH. In addition, the *ALK* FISH scoring criteria could fail for heterogeneous cases aggravated by the fact that tissue sections are used where nuclei are typically clipped.

Case 49 with potential detection of *EZR-ROS1* rearrangement but negative Ros1 IHC could be most likely false positive. However, since we did not perform a dilution series of RNA of a *ROS1*rearranged cell line and tissue for FISH testing was not available, we cannot completely solve this case.

We observed RNA concentrations from 11.2 to 152 ng/μl although three cores had been taken and RNA was extracted from each core. The varying RNA amounts are most likely due to differences in the thickness of tissue blocks and cellularity. Although only LUADs with tumor content ≥ 60% were selected, some cores probably contained less cells in deeper areas of the core.

In summary, targeted NGS for fusion detection is a more robust and reliable method in detection of *ALK* and other targetable rearrangements compared to single target assays such as FISH. In addition, turnaround time, reproducibility and superior cost efficiency favor the use of targeted NGS compared with conventional single target assays for fusion detection.

## 5 Conclusion

Detection of targetable fusion genes is crucial for appropriate clinical action. We experienced the RNA-based OFA panel as a reliable and accurate tool in comparison with standard single target assays like FISH and IHC. Especially borderline cases can benefit from the added value of NGS testing.

## Conflict of interest

The authors declare that no conflict of interest exists.
